# Associations of genetically predicted iron status with 24 gastrointestinal diseases and gut microbiota: a Mendelian randomization study

**DOI:** 10.3389/fgene.2024.1406230

**Published:** 2024-08-07

**Authors:** Tao Su, Xiang Peng, Ying Gan, Hongzhen Wu, Shulin Ma, Min Zhi, Yi Lu, Shixue Dai, Jiayin Yao

**Affiliations:** ^1^ Department of Gastroenterology, Guangdong Provincial Key Laboratory of Colorectal and Pelvic Floor Disease, The Sixth Affiliated Hospital, Sun Yat-Sen University, Guangzhou, China; ^2^ Biomedical Innovation Center, The Sixth Affiliated Hospital, Sun Yat-Sen University, Guangzhou, China; ^3^ Department of Anesthesiology, The Affiliated TCM Hospital of Guangzhou Medical University, Guangzhou, China; ^4^ Department of Gastroenterology, Guangdong Provincial Geriatrics Institute, National Key Clinical Specialty, Guangdong Provincial People’s Hospital, Guangdong Academy of Medical Sciences, Guangzhou, China

**Keywords:** iron status, gastrointestinal diseases, gut microbiota, Mendelian randomization, liver cancer

## Abstract

**Background:**

Iron status has been implicated in gastrointestinal diseases and gut microbiota, however, confounding factors may influence these associations.

**Objective:**

We performed Mendelian randomization (MR) to investigate the associations of iron status, including blood iron content, visceral iron content, and iron deficiency anemia with the incidence of 24 gastrointestinal diseases and alterations in gut microbiota.

**Methods:**

Independent genetic instruments linked with iron status were selected using a genome-wide threshold of *p* = 5 × 10−6 from corresponding genome-wide association studies. Genetic associations related to gastrointestinal diseases and gut microbiota were derived from the UK Biobank, the FinnGen study, and other consortia.

**Results:**

Genetically predicted higher levels of iron and ferritin were associated with a higher risk of liver cancer. Higher levels of transferrin saturation were linked to a decreased risk of celiac disease, but a higher risk of non-alcoholic fatty liver disease (NAFLD) and liver cancer. Higher spleen iron content was linked to a lower risk of pancreatic cancer. Additionally, higher levels of liver iron content were linked to a higher risk of NAFLD and liver cancer. However, certain associations lost their statistical significance upon accounting for the genetically predicted usage of cigarettes and alcohol. Then, higher levels of iron and ferritin were associated with 11 gut microbiota abundance, respectively. In a secondary analysis, higher iron levels were associated with lower diverticular disease risk and higher ferritin levels with increased liver cancer risk. Higher levels of transferrin saturation were proven to increase the risk of NAFLD, alcoholic liver disease, and liver cancer, but decrease the risk of esophageal cancer. MR analysis showed no mediating relationship among iron status, gut microbiota, and gastrointestinal diseases.

**Conclusion:**

This study provides evidence suggesting potential causal associations of iron status with gastrointestinal diseases and gut microbiota, especially liver disease.

## Introduction

Maintaining iron homeostasis is crucial for the survival of most living organisms. As a nutritionally essential trace element, iron can impact vital bodily functions such as blood production, oxygen transportation, and various metabolic pathways (McClung, 2019). Most of the iron in the human body is present in the hemoglobin of red blood cells. The concentration of iron in the cells is approximately 1 mg per milliliter, and the total content ranges from 2 to 2.5 g (Nemeth and Ganz, 2023). Among the organs, the liver and spleen have the highest content, followed by the kidney, heart, pancreas, and brain. Due to the low bioavailability of iron in foods, this nutrient deficiency is a worldwide public health problem (Yang et al., 2023). The body, however, cannot effectively metabolize excessive iron due to limited excretion pathways. Consequently, the consumption of an excessive amount of iron or accidental ingestion can also lead to iron poisoning (Jacobs et al., 1965).

Gastrointestinal diseases are also a widespread global health problem that imposes a significant economic burden worldwide (Peery et al., 2019). The annual healthcare expenditure for gastrointestinal diseases in the United States amounts to 135.9 billion dollars, surpassing that of other prevalent illnesses, and is projected to continue its upward trend (Peery et al., 2022). Consequently, elucidating the pathogenesis of gastrointestinal diseases and developing corresponding interventions or preventive measures are imperative for reducing disease incidence and alleviating the associated burden. Epidemiological studies have found an association between iron status and the risk of multiple sites of gastrointestinal disease, including non-alcoholic fatty liver disease (NAFLD), colorectal cancer, and inflammatory bowel disease (Mayneris-Perxachs et al., 2021; Phipps et al., 2021; Xu et al., 2021). However, the association between iron status and other prevalent gastrointestinal diseases remains insufficiently explored. In addition, due to the inherent limitations of observational studies, like susceptibility to reverse causality and confounding factors, establishing a definitive causal association between iron status and gastrointestinal diseases remains uncertain.

Mendelian randomization (MR) is an epidemiological approach that employs genetic variation as an instrumental variable to infer causal associations between exposure and outcome (Hemani et al., 2017; Bowden and Holmes, 2019). Given that genetic variation is determined randomly before birth and remains unaffected by factors such as age or disease, it mitigates confounding variables and reverses causation. Although previous MR studies have identified an association between iron status and several gastrointestinal diseases, the association with other gastrointestinal conditions such as cirrhosis remains inconclusive and conclusions have varied due to disparate database sources utilized (Liu et al., 2022; Wang et al., 2022). Additionally, the association between visceral iron content and gastrointestinal disease and the impact of iron status on gut microbiota remains inadequately elucidated in previous MR studies. Here, we performed MR to investigate the associations of iron status, including blood iron content (iron, ferritin, transferrin saturation, and transferrin), visceral iron content (spleen, liver, and pancreas), and iron deficiency anemia with the risk of 24 gastrointestinal diseases and 196 gut microbiota.

## Methods

### Study design

**FIGURE 1:**
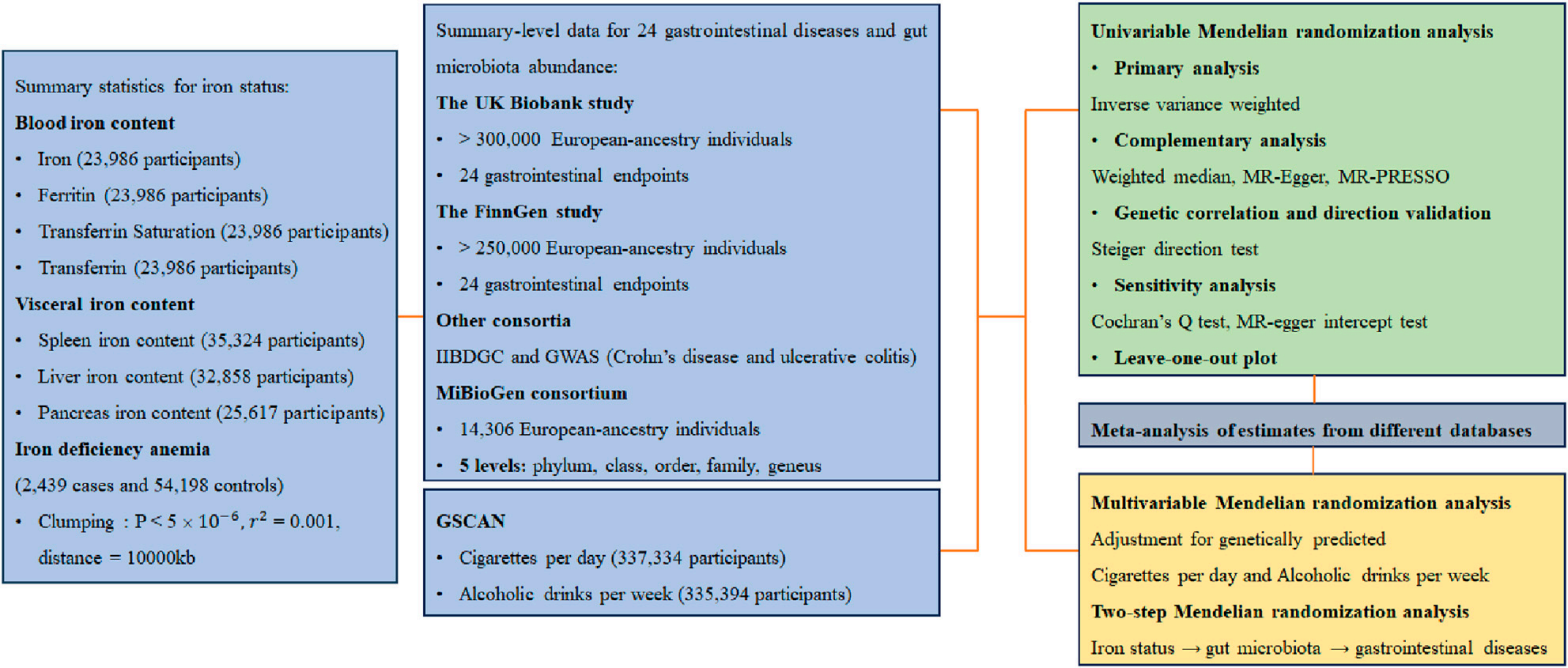
Study design GIS, Genetics of Iron Status Consortium; IIBDGC, International Inflammatory Bowel Disease Genetics Consortium; GWAS, datasets of Katrina M de Lange et al., PubMed ID: 28067908; GSCAN, GWAS and Sequencing Consortium of Alcohol and Nicotine use; MR, Mendelian randomization; MR-PRESSO, Mendelian randomization pleiotropy residual sum and outlier.

The visual representation of the study design can be observed in Figure 1. This study is based on publicly available genome-wide association studies (GWASs); therefore, ethical approval was exempted (Supplementary Table S1).

### Data sources

We acquired summary-level blood iron content data from a meta-analysis by the Genetics of Iron Status Consortium (GIS), which comprised 23,986 European ancestry individuals (Benyamin et al., 2014). The validation cohort data for iron and ferritin were acquired from a study by Dennis JK et al. (Dennis et al., 2021)

For visceral iron content, genetic associations with liver and pancreas iron content were obtained from a GWAS of about 30,000 European individuals in which the combination of deep learning and efficient image processing of magnetic resonance imaging measured iron content (Liu et al., 2021). Spleen iron content was measured similarly, and the corresponding GWAS data were derived from the UK Biobank study involving 35,324 participants (Sorokin et al., 2022). The GWAS of Guindo-Martínez M et al. provided genetic associations with iron deficiency anemia with 2,439 cases and 54,198 controls (Guindo-Martínez et al., 2021).

Summary-level statistics on gastrointestinal diseases were extracted from the UK Biobank and the FinnGen consortium data freeze 9 results (Sudlow et al., 2015). Additionally, summary-level statistics of gastrointestinal diseases in individuals of European ancestry were obtained from the GWASs by Sakaue et al. within the UK Biobank (Sakaue et al., 2021). We additionally extracted two sets of summary-level data on Crohn’s disease (CD) and ulcerative colitis (UC) from the International Inflammatory Bowel Disease Genetics Consortium (IIBDGC) and a GWAS meta-analysis of Katrina M. de Lange Team (Liu et al., 2015; de Lange et al., 2017). Detailed descriptions of outcomes are shown in Supplementary Table S1 and Supplementary Table S2. The summary data of gut microbiota were obtained from a multi-ethnic meta-analysis of GWASs, encompassing 18,340 individuals across 24 cohorts (Kurilshikov et al., 2021). The microbial composition was analyzed by focusing on three distinct variable regions within the 16S rRNA gene. Following the exclusion of 15 unidentified taxa, a comprehensive set of 196 taxa was considered for the MR analysis.

### Selection of genetic instrument

We extracted single-nucleotide polymorphisms (SNPs) linked to iron status using a genome-wide threshold of *p* = 5 × 10^−6^ from the GWASs mentioned above. After clumped within a genomic region of 10,000 kb and a linkage disequilibrium (LD) r^2^ threshold of 0.001, independent SNPs were employed as genetic instruments in MR analysis. The utilization of proxy SNPs is not applicable in this study. Supplementary Table S3 provides comprehensive details on the genetic tools employed in this study. In multivariate MR analysis, the genome-wide threshold was set at *p* = 5 × 10^−8^ for genetically predicted tobacco and alcohol consumption. However, in the two-step MR analysis, the threshold of 1 × 10^−5^ was used to extract instrumental variables representing gut microbiota for obtaining sufficient SNPs.

### Statistical analysis

We evaluated the genetic instruments’ effectiveness by computing the F-statistic for each SNP. SNPs with an F-statistic lower than 10 were considered inadequate instruments and were not included in the analysis (Pierce et al., 2011; Pierce and Burgess, 2013). The primary method employed to evaluate the association between iron status and outcomes was inverse variance weighted (IVW). The IVW method integrates the Wald ratio of single SNPs, assuming that instrumental variables exert a causal effect on the outcome exclusively through the exposure and not via any alternative pathway. Three analyses, including weighted median, MR Egger regression, and MR pleiotropy residual sum and outlier (MR-PRESSO) were performed as additional analytical methods. The weighted median method can yield a robust estimate if the proportion of valid instrumental variables exceeds 50%. In contrast to the IVW method, MR Egger regression incorporates the inclusion of an intercept in the model to effectively examine and control for pleiotropy, as it is expected that when the exposure effects are null, the outcome should also be null. MR-PRESSO comprises three components: identification of horizontal pleiotropy, correction through outlier elimination, and assessment of significant differences in causal estimation before and after addressing abnormal values (Verbanck et al., 2018). It was employed to identify significant outliers and address potential horizontal pleiotropic effects by removing these outliers. Furthermore, the meta-analysis was utilized to combine estimates from various sources, employing the random effects model when I^2^ exceeded 60%, and alternatively using the fixed effects model.

The Steiger test was conducted to identify and address potential issues of reverse causality, ensuring the association between exposure and outcomes in a causal manner is accurate. The Cochrane Q test assessed heterogeneity, while the MR Egger intercept test was employed for investigating horizontal pleiotropy (Bowden et al., 2015; Greco et al., 2015). We performed a multivariate MR analysis to assess whether the correlation between iron status and gastrointestinal disease remains significant even after accounting for genetically predicted tobacco and alcohol consumption, which are strongly linked to gastrointestinal disease. The association was considered potentially significant if the *p*-value was below 0.05, but with a Benjamini-Hochberg-adjusted *p*-value larger than 0.05, suggesting the need for further investigations to validate these findings. On the other hand, an association was deemed statistically significant when the Benjamini-Hochberg-adjusted *p*-value was below 0.05. Finally, we performed a two-step MR analysis to test whether gut microbiota mediates the genetic association between iron status and gastrointestinal diseases. The TwoSampleMR package (version 0.5.7) was utilized for performing the MR analysis in R software (version 4.2.3).

## Results

The F-statistic of all SNP was larger than 10, ranging from 14.78 to 1429.33 indicating robust efficacy of the genetic tools employed (Supplementary Table S3).

### Blood iron content

**FIGURE 2:**
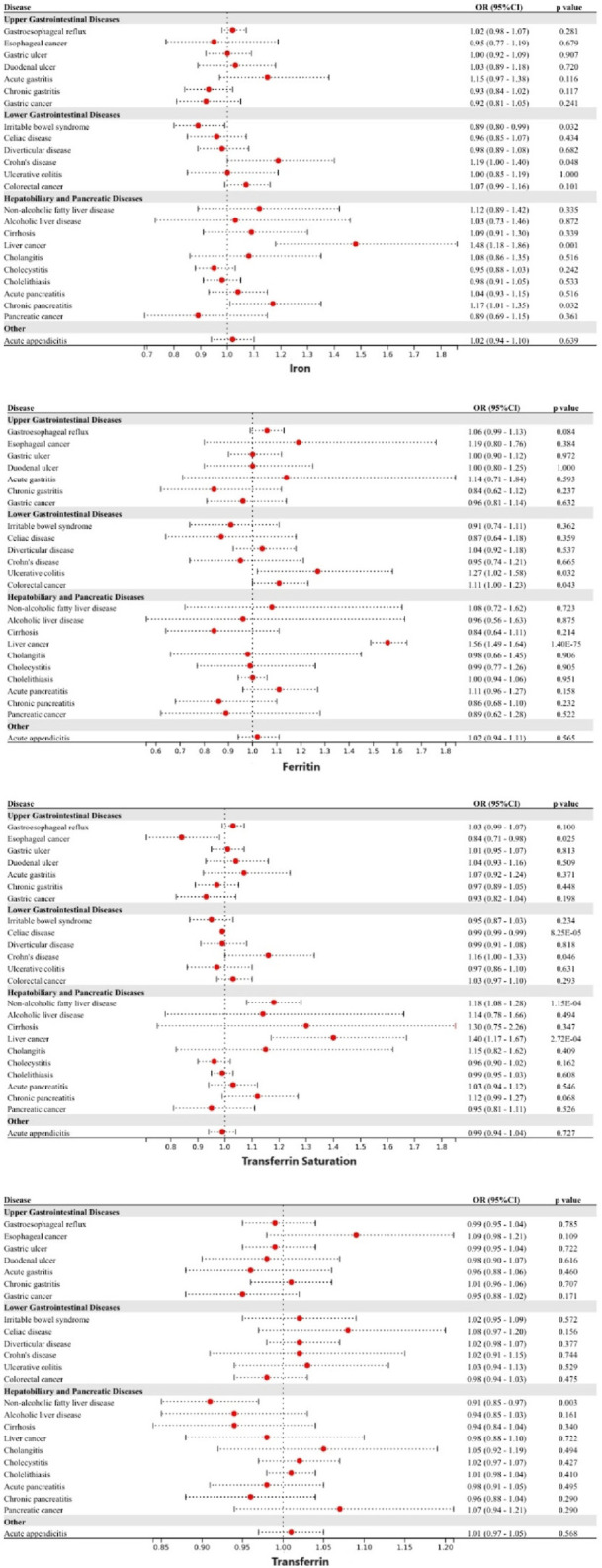
Associations of genetically-predicted blood iron content with 24 gastrointestinal diseases. OR, odds ratio; CI, confidence interval.

Genetically predicted higher levels of iron were linked to a decreased risk of irritable bowel syndrome, but a higher risk of CD, liver cancer, and chronic pancreatitis. Genetically predicted higher levels of ferritin were linked to a higher risk of UC, colorectal cancer, and liver cancer. Genetically predicted higher levels of transferrin saturation were linked to a lower risk of esophageal cancer and celiac disease, but a higher risk of CD, NAFLD, and liver cancer. Finally, a decreased risk of NAFLD was observed in individuals with genetically predicted higher levels of transferrin (Figure 2). After the Benjamini–Hochberg adjustment, genetically predicted higher levels of iron (odds ratio [OR]: 1.48, 95% Confidence Interval [CI]: 1.18—1.86) and ferritin (OR: 1.56, 95%CI: 1.49—1.64) were linked to a higher risk of liver cancer. Genetically predicted higher levels of transferrin saturation were linked to a decreased risk of celiac disease (OR: 0.99, 95% CI: 0.99—0.99), but a higher risk of NAFLD (OR: 1.18, 95%CI: 1.08—1.28) and liver cancer (OR: 1.40, 95%CI: 1.17—1.67). These associations were consistent in all additional analytical methods. No heterogeneity and pleiotropy were detected, and MR-PRESSO did not find any outliers. Genetically predicted blood iron content did not show any significant association with the other gastrointestinal diseases under investigation. The associations between liver cancer with iron and transferrin saturation remained consistent after adjustment for genetically predicted tobacco and alcohol consumption, while the other genetic associations were weakened or insignificant.

### Visceral iron content

**FIGURE 3:**
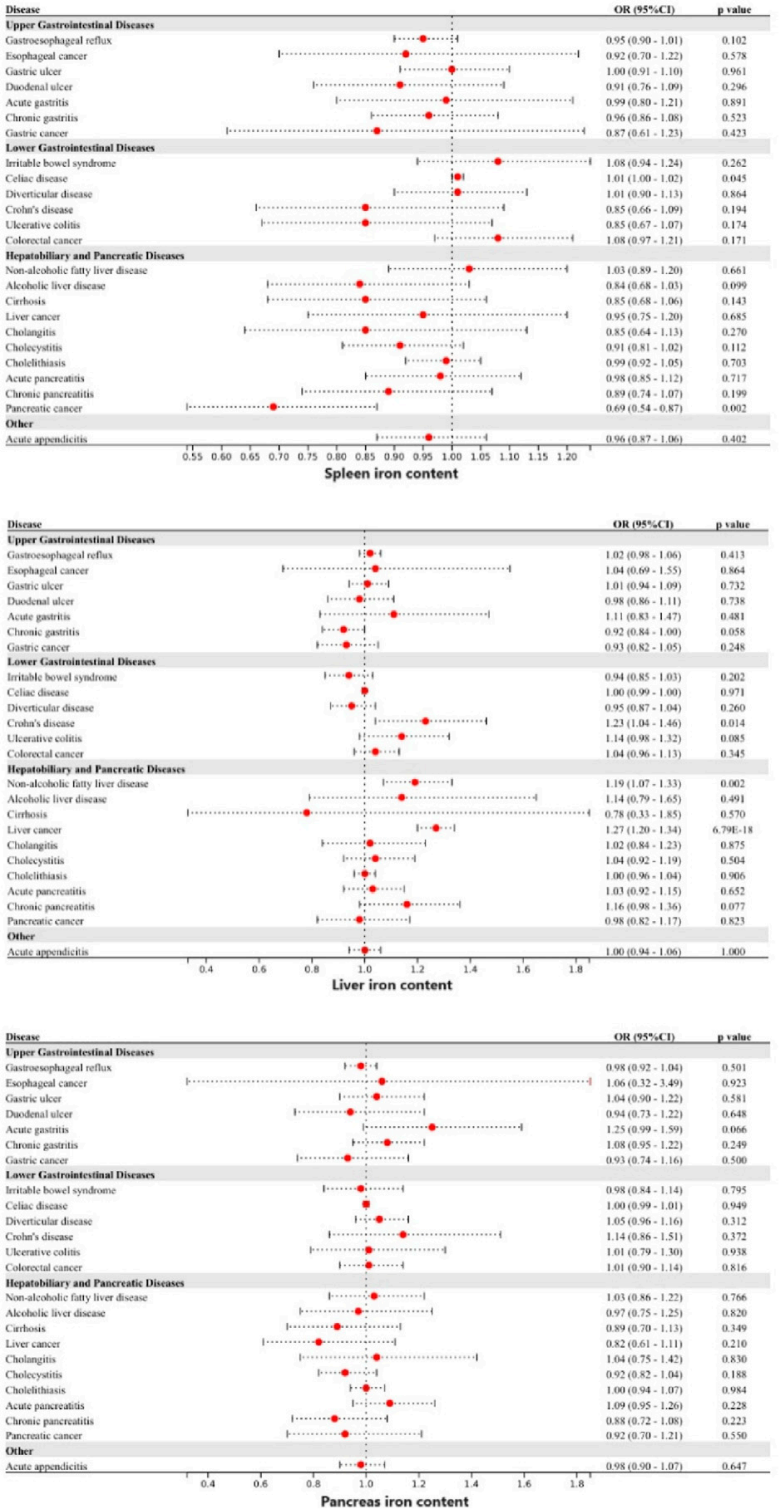
Associations of genetically-predicted visceral iron content with 24 gastrointestinal diseases. OR, odds ratio; CI, confidence interval.

Genetically predicted higher levels of spleen iron content were linked to a higher risk of celiac disease and a lower risk of pancreatic cancer. Genetically predicted increased levels of liver iron content were linked to a higher risk of CD, NAFLD, and liver cancer. Genetically predicted pancreas iron content was not associated with any of the 24 gastrointestinal diseases studied (Figure 3). Following adjustment for multiple comparisons, the associations between liver iron content with NAFLD (OR: 1.19, 95%CI: 1.07—1.33) and liver cancer (OR: 1.27, 95%CI: 1.20—1.34) persisted. The associations remained consistent in the other three methods. Higher genetically predicted spleen iron content was linked to a lower risk of pancreatic cancer (OR: 0.69, 95%CI: 0.54—0.87). The absence of heterogeneity and pleiotropy was not observed, and no outliers were detected by MR-PRESSO. However, after adjustment for genetically predicted tobacco and alcohol consumption, the genetic associations above were all weakened.

### Iron deficiency anemia

**FIGURE 4:**
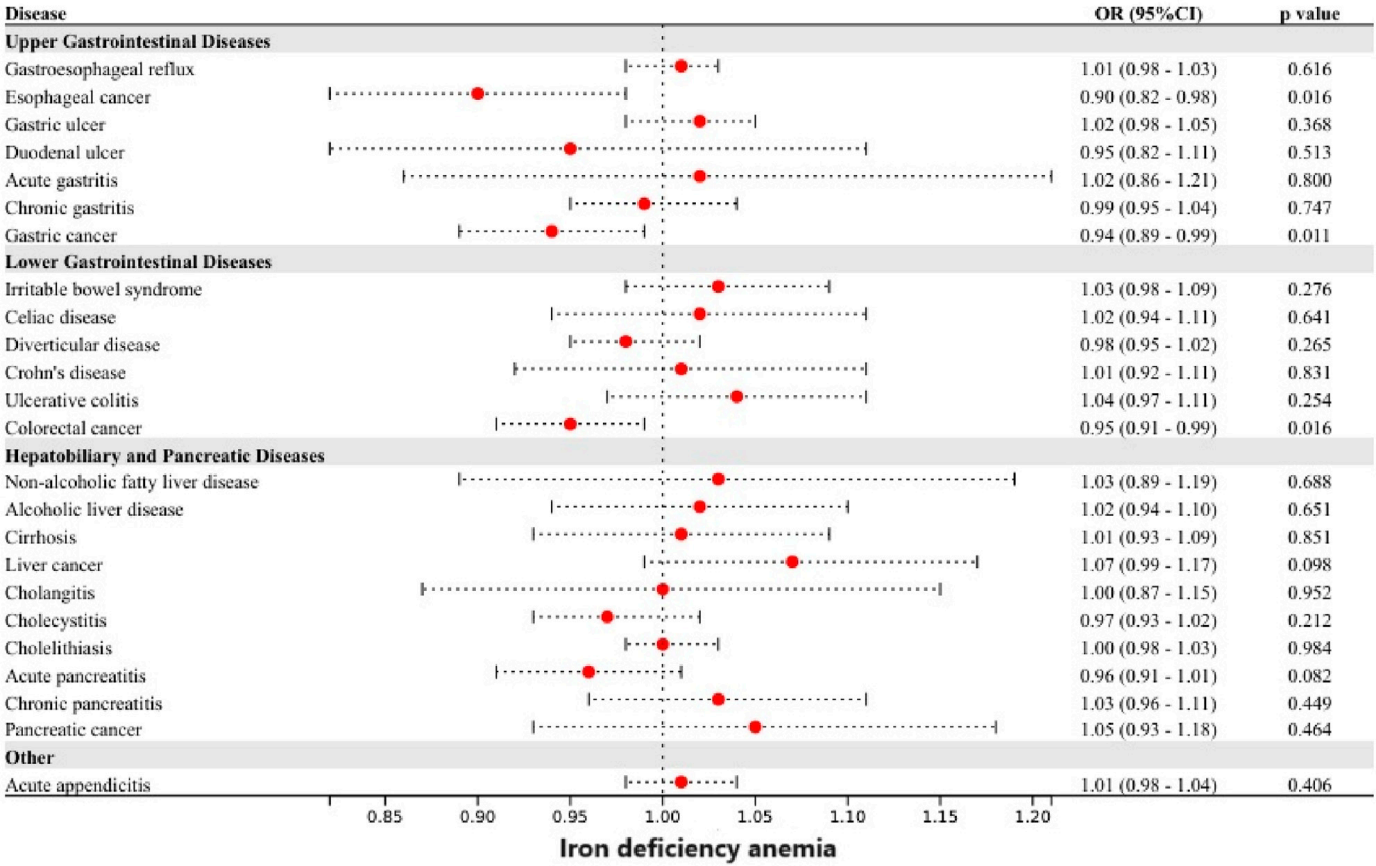
Associations of genetically-predicted iron deficiency anemia with 24 gastrointestinal diseases. OR, odds ratio; CI, confidence interval.

Genetically predicted iron deficiency anemia was linked to a lower risk of esophageal cancer, gastric cancer, and colorectal cancer (Figure 4). However, after correction for multiple comparisons, all remained suggestive. Heterogeneity was detected in the examination of colorectal cancer within the UK Biobank study (Cochran’s Q *p*-value <0.05), however, no exceptional data point was identified by MR-PRESSO.

### Gut microbiota abundance

Genetically predicted iron levels were significantly associated with the relative abundance of 11 taxonomic groups, including 2 classes, 1 family, 7 genera, and 1 phylum. Specifically, these taxa were Alphaproteobacteria id.2379, Gammaproteobacteria id.3303, Methanobacteriaceae id.121, Anaerotruncus id. 2054, Christensenellaceae R 7group id.11283, Family XIII AD3011 group id.11293, Paraprevotella id.962, Ruminiclostridium9 id.11357, Ruminococcaceae NK4A214 group id.11358, Ruminococcaceae UCG010 id.11367, and Tenericutes id.3919 (Supplementary Table S17).

Similarly, genetically predicted ferritin levels were associated with the abundance of 1 class, 2 family, 7 genera, and 1 order. They were Deltaproteobacteria id.3087, Acidaminococcaceae id.2166, Desulfovibrionaceae id.3169, Butyrivibrio id. 1993, Christensenellaceae R 7group id.11283, Odoribacter id.952, Ruminococcaceae NK4A214 group id.11358, Ruminococcaceae UCG005 id.11363, Ruminococcaceae UCG010 id.11367, Sellimonas id.14369, and Rhodospirillales id.2667 (Supplementary Table S18). After adjustment for multiple comparisons, higher genetically predicted iron levels were linked to lower flora abundance of Family XIII AD3011 group id.11293 (Beta: −0.151, 95%CI: −0.228 to −0.074, Benjamini–Hochberg adjusted *p* = 0.015) and Ruminococcaceae NK4A214 group id.11358 (Beta: −0.15, 95%CI: −0.227 to −0.072, Benjamini–Hochberg adjusted *p* = 0.015).

### Secondary analysis

In the analysis of blood iron content, we found that several SNPs were associated with multiple markers of iron status, such as rs1800562. Secondary analyses were performed by including these SNPs in the main analysis of the single marker with the strongest association. The results showed that higher levels of iron were associated with lower diverticular disease risk (OR: 0.86, 95%CI: 0.74—0.99) and higher levels of ferritin with increased liver cancer risk (OR: 1.38, 95%CI: 1.04—1.81). Higher levels of transferrin saturation were proven to increase the risk of NAFLD (OR: 1.18, 95%CI: 1.08—1.29), alcoholic liver disease (OR: 1.18, 95%CI: 1.00—1.39), and liver cancer (OR: 1.42, 95%CI: 1.17—1.72), but decrease the risk of esophageal cancer (OR: 0.83, 95%CI: 0.71—0.97). (Supplementary Table S20–S23).

### Two-step MR analysis

Based on the results of the above MR analysis, we employed a two-step MR analysis to investigate the presence of the following genetic associations: iron status → gut microbiota → gastrointestinal diseases. According to the data source (UK Biobank or FinnGen study), we performed two sets of analyses, both of which showed that the gut microbiota was not a mediator of the genetic association between iron status and gastrointestinal diseases (Supplementary Table S24).

## Discussion

This is the first comprehensive MR analysis to investigate the associations of iron status, including blood iron content, visceral iron content, and iron deficiency anemia with the risk of 24 gastrointestinal diseases and the changes in gut microbiota. The study found that genetically predicted iron status was linked to a variety of gastrointestinal diseases and gut microbiota abundance. After adjustment, the associations appeared to exist primarily with liver disease, especially liver cancer.

In a typical diet, only 10% of iron is absorbed in the small intestine and residual iron is present in the gastrointestinal (GI) tract (Waldvogel-Abramowski et al., 2014). Excess iron in the GI tract may lead to increased proliferation and transformation of intestinal cells into tumor cells. The augmentation of dietary iron has been substantiated through animal experiments to induce the proliferation of crypt cells in the large intestine and elevate the incidence rate of colon tumors (Siegers et al., 1992; Lund et al., 1998). Although our study did not demonstrate a substantial association between blood iron content and colorectal cancer, there is indeed an association observed with certain conditions such as ulcerative colitis. It may be that iron-induced reactive oxygen species (ROS) increase the oxidative stress response in the GI tract, which leads to GI inflammation (Aghdassi et al., 2001; Carrier et al., 2002; Verma and Cherayil, 2017).

**FIGURE 5:**
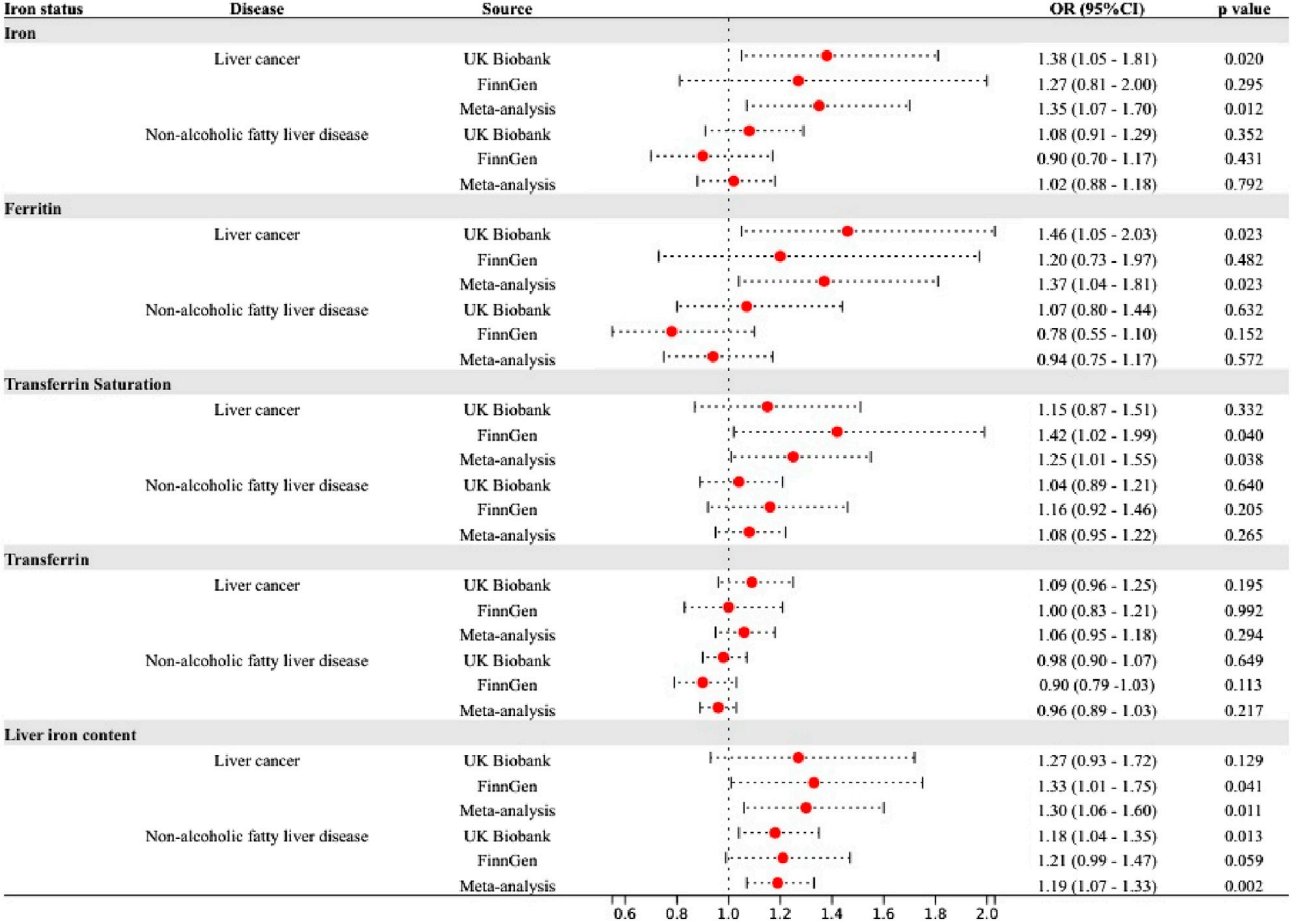
Associations of genetically-predicted iron status with 24 gastrointestinal diseases excluding SNP rs1800562. OR, odds ratio; CI, confidence interval.

The liver serves as the primary organ for iron metabolism and is also subject to the influence of iron homeostasis (Yu et al., 2020). A study based on autopsy demonstrated that prolonged intravenous iron administration, even at the lowest recommended dosage, led to increased hepatic iron deposition (Akatsu et al., 2020). Patients with iron overload are at increased risk for liver disease. The results of our study are in strong agreement with previous observational studies. In a recent meta-analysis, the highest serum ferritin and serum iron levels showed an elevated risk of liver cancer by 1.5 times and 2.5 times, respectively, compared with the lowest group (Tran et al., 2019). In addition, reaching a transferrin saturation level of 60% or higher, as opposed to less than 50%, was found to be linked with a substantial 5.9-fold rise in the likelihood of developing liver cancer (Ellervik et al., 2012). A meta-analysis showed that dietary iron intake was higher in Asian patients with NAFLD than in healthy subjects (Li et al., 2021). Experimental studies have reported that iron-induced ROS and lipid peroxidation cause damage to hepatocyte organelles (Ramm and Ruddell, 2005). ROS induce an upregulation of the oxidative stress response, resulting in DNA damage and protein modification. The occurrence of DNA damage caused by oxidative stress can lead to genomic instability and subsequently contribute to carcinogenesis (Reuter et al., 2010; Zeng et al., 2023). Cytotoxic by-products of lipid peroxidation impair cellular function, protein synthesis, and DNA integrity. Reactive oxygen intermediates can induce the formation of mutagenic adducts in DNA, leading to DNA strand unwinding and breakage (Kew, 2009). The above mechanism can lead to liver cancer. Iron overload in hepatic stellate cells leads to excessive production of ROS, thereby promoting fibrotic activation in NAFLD patients (Gao et al., 2022). Early animal experiments showed that the increase of liver iron promotes the significant upregulation of seven enzyme transcripts in the cholesterol biosynthesis pathway, which may promote the occurrence and development of fatty liver disease or lipotoxicity (Graham et al., 2010). In addition, iron may aggravate hepatic insulin resistance, which leads to NAFLD (Angulo et al., 1999; Altamura et al., 2021). It has also been reported that excessive iron-induced liver ferroptosis played a key role in liver dysfunction and injury in different mouse models of liver fibrosis/cirrhosis (Kong et al., 2019; Wang et al., 2019). Significantly, the variant rs1800562 in the HFE gene, commonly referred to as C282Y, is associated with a severe manifestation of Hemochromatosis. This SNP is associated with reduced hepcidin levels, resulting in increased iron absorption and parenchymal deposition in the liver (iron overload). A 2016 meta-analysis conducted by Qing et al. found evidence suggesting that individuals with HFE C282Y polymorphisms may have a higher genetic predisposition to developing NAFLD and liver cancer, while the risk of cirrhosis does not appear to be affected (Ye et al., 2016). Following the exclusion of rs1800562, the results revealed weakened or nonexistent associations between iron status and NAFLD as well as liver cancer (Figure 5). Besides, previous MR Studies provided evidence supporting the causal association of smoking and alcohol consumption with a variety of gastrointestinal diseases, including liver cancer and NAFLD (Yuan et al., 2023). In the present study, we corrected the bias of smoking and alcohol consumption using multivariate MR analysis. Despite observing a weakened association between iron status and other diseases, our findings still support a causal association between iron status and liver cancer, highlighting the independent role of iron in liver cancer development. However, it is important to mention that our research also resulted in the surprising finding of a correlation between spleen iron content and pancreatic cancer. The specific mechanism still needs to be further studied.

A study in 2021 also revealed a significant interaction between the gut microbiota, iron status, and hepatic lipid accumulation (Mayneris-Perxachs et al., 2021). Despite discrepancies in metagenomics findings, both studies have consistently demonstrated the impact of iron on the gut microbiome composition. Similarly, an increase in serum ferritin levels was found to be linked with the accumulation of lipids in the liver and alterations in both the composition and functionality of the gut microbiota. The supplementation of dietary iron in rats was also associated with an increase in Coprococcus caecal content. This particular bacterium was hypothesized to contribute towards the mediation of oxidative stress and histopathological alterations observed in the liver of these subjects (Fang et al., 2018). Excessive dietary iron intake was proved to stimulate the permeability of the intestinal barrier, resulting in luminal bacterial leakage. Consequently, epithelial cells responded by releasing an increased amount of secretory leukocyte protease inhibitors (SLPI) to combat bacterial leakage and restrict inflammation (Liu et al., 2023). As a pro-tumor factor, upregulated SLPI promotes colorectal tumorigenesis through activation of the MAPK signaling pathway. SLPI could also expedite tumor progression and facilitate tumor metastasis (Sugino et al., 2007; Wei et al., 2020). Our results showed that higher levels of iron reduced the abundance of Ruminococcaceae NK4A214 group id.11358. An imbalance in iron metabolism-related flora has been observed in patients with liver cirrhosis, and Ruminococcaceae can counteract pro-inflammatory factors and exert a protective role in the disease (Iebba et al., 2018). In animal experiments, increased Ruminococcaceae was associated with the beneficial effects of deferiprone in the treatment of iron overload mice (Sriwichaiin et al., 2022). Although our analysis did not reveal a mediating effect of gut microbiota on the genetic association between iron status and gastrointestinal diseases, it is important to note that our study was limited by data availability. Specifically, we analyzed only 197 gut microbiota, which may have excluded many other relevant microbiota from our investigation.

Our MR study comprehensively analyzed the genetic association between iron status, gastrointestinal diseases, and gut microbiota. Anemia is a prevalent condition in clinics, with iron deficiency anemia being the predominant form. Oral or intravenous administration of iron is commonly employed as a therapeutic approach. Current guidelines recommend the continuation of iron therapy for 4–6 months after hemoglobin normalization to facilitate replenishment of iron stores. However, our study showed that excessive iron status is associated with a higher risk of various diseases, especially liver cancer. As mentioned above, transferrin saturation of 60% or higher is associated with a significant increase in the likelihood of liver cancer (Ellervik et al., 2012). Therefore, we believe that monitoring transferrin saturation during iron therapy administration may constitute an indispensable preventive measure in individuals at high risk. For patients with iron overload, Ruminococcaceae may be an effective supplement for deferiprone therapy. Although the DrugBank platform currently does not provide information on Ruminococcaceae-related products, advancements in medical technology, such as fecal bacteria transplantation, have made it possible to utilize intestinal flora for adjunctive treatment of iron overload in the future (Ooijevaar et al., 2019).

In conclusion, our study suggested that genetically predicted iron status was linked to a broad range of gastrointestinal diseases and gut microbiota abundance, which may cause clinical attention to iron status in high-risk groups of the disease and develop corresponding preventive measures.

Our study had several limitations. Initially, we exclusively utilized data from European populations to mitigate the influence of population stratification bias. However, this choice restricts the applicability of our results to different populations. Simultaneously, due to a limited number of cases of certain gastrointestinal diseases, our statistical power is diminished. Low statistical performance may lead to false negatives or false positives. Additionally, the primary limitation lies in the sample overlap between the visceral iron content dataset and the outcome data. Although a sample overlap rate of up to 10% still provides a conclusion with 90% power of the full-sample instrumental variable analysis in MR analysis, it undeniably affects the reliability of the research findings (Pierce and Burgess, 2013). Therefore, further studies are needed to validate our findings.

## Data Availability

The original contributions presented in the study are included in the article/Supplementary Material, further inquiries can be directed to the corresponding authors.
